# An *ADAM9* mutation in canine cone-rod dystrophy 3 establishes homology with human cone-rod dystrophy 9

**Published:** 2010-08-11

**Authors:** Orly Goldstein, Jason G. Mezey, Adam R. Boyko, Chuan Gao, Wei Wang, Carlos D. Bustamante, Lynne J. Anguish, Julie Ann Jordan, Susan E. Pearce-Kelling, Gustavo D. Aguirre, Gregory M. Acland

**Affiliations:** 1 Baker Institute for Animal Health, Cornell University College of Veterinary Medicine, Ithaca, NY; 2 Department of Biological Statistics and Computational Biology, Cornell University, Ithaca, NY; 3 Department of Genetic Medicine, Weill Cornell Medical College, NY, NY; 4 Department of Genetics, Stanford University School of Medicine, Stanford, CA; 5 Microarray Core Facility, Cornell University, Ithaca, NY; 6 Optigen, LLC, Ithaca, NY; 7 School of Veterinary Medicine, University of Pennsylvania, Philadelphia, PA

## Abstract

**Purpose:**

To identify the causative mutation in a canine cone-rod dystrophy (crd3) that segregates as an adult onset disorder in the Glen of Imaal Terrier breed of dog.

**Methods:**

Glen of Imaal Terriers were ascertained for crd3 phenotype by clinical ophthalmoscopic examination, and in selected cases by electroretinography. Blood samples from affected cases and non-affected controls were collected and used, after DNA extraction, to undertake a genome-wide association study using Affymetrix Version 2 Canine single nucleotide polymorphism chips and 250K Sty Assay protocol. Positional candidate gene analysis was undertaken for genes identified within the peak-association signal region. Retinal morphology of selected crd3-affected dogs was evaluated by light and electron microscopy.

**Results:**

A peak association signal exceeding genome-wide significance was identified on canine chromosome 16. Evaluation of genes in this region suggested A Disintegrin And Metalloprotease domain, family member 9 (*ADAM9)*, identified concurrently elsewhere as the cause of human cone-rod dystrophy 9 (CORD9), as a strong positional candidate for canine crd3. Sequence analysis identified a large genomic deletion (over 20 kb) that removed exons 15 and 16 from the *ADAM9* transcript, introduced a premature stop, and would remove critical domains from the encoded protein. Light and electron microscopy established that, as in *ADAM9* knockout mice, the primary lesion in crd3 appears to be a failure of the apical microvilli of the retinal pigment epithelium to appropriately invest photoreceptor outer segments. By electroretinography, retinal function appears normal in very young crd3-affected dogs, but by 15 months of age, cone dysfunction is present. Subsequently, both rod and cone function degenerate.

**Conclusions:**

Identification of this *ADAM9* deletion in crd3-affected dogs establishes this canine disease as orthologous to CORD9 in humans, and offers opportunities for further characterization of the disease process, and potential for genetic therapeutic intervention.

## Introduction

Cone-rod dystrophies are severe hereditary retinal diseases characterized by primary dysfunction and loss of cone photoreceptors accompanying or preceding that of rods. The typical age of clinical onset in affected humans ranges from early to late adulthood; autosomal dominant, recessive, and X-linked forms of the disease occur. Multiple mapped human loci are recognized, including over a dozen causative genes (RETNET). A clinically similar disorder, termed canine cone-rod dystrophy 3 (crd3), segregates in the Irish Glen of Imaal Terrier (GIT) breed of dog as an adult onset trait of previously undetermined mode of inheritance. This disease becomes evident ophthalmoscopically in affected dogs as young as 3 years of age, and progresses to end-stage retinal degeneration over several years. Concomitantly, the dogs develop visual problems; these usually manifest first as difficulties avoiding obstacles in dim light, and worsen over several years to apparent total blindness. Its mode of inheritance has been difficult to establish from natural populations, because of the multiple inbreeding loops in natural pedigrees. Previous candidate gene studies have excluded unc-119 homolog (*C. elegans*) (*UNC119 *[*HGR4*])*,* cone-rod homeobox (*CRX*)*,* peripherin 2, retinal degeneration, slow (*PRPH2*), interphotoreceptor matrix proteoglycan 1 (*IMPG-1*)*,* tissue inhibitor of metalloproteinase 3 (*TIMP3*), and retinitis pigmentosa GTPase regulator interacting protein (*RPGRIP1*) as the causative loci (unpublished data).

The GIT is a numerically small breed originating from the Wicklow Mountains region of Ireland. Although the breed has been known internationally since the 19th century, and was first registered in 1934, the present-day population derives from a very small number of founders from the 1970s. It is thus a genetic isolate, with a small gene pool, significant inbreeding, and pedigrees with multiply overlapping generations. This population structure predicts extensive linkage disequilibrium flanking the mutation site, and creates an ideal opportunity to map the causative locus by a genome-wide association study (GWAS), as has been demonstrated and exploited for other canine traits [1–8].

The present study reports results of a GWAS that found significant association to crd3 on canine chromosome 16 (CFA16), and led to identification of a deletion mutation in the canine *ADAM9* gene that cosegregates with the disease. The mutation removes approximately 23 kb of genomic sequence, including exons 15 and 16, and results in a premature stop codon in exon 17. The mutant protein translated from this transcript is predicted to be truncated, lacking the last 287 amino acids of the C-terminus, part of the cysteine-rich domain, the complete epidermal growth factor (EGF)-like domain, the transmembrane domain, and the cytoplasmic tail. The association of this deletion mutation in canine *ADAM9* with crd3 establishes that this canine disease is orthologous to human CORD9 [9].

## Methods

### Animal use

All procedures involving animal care were conducted in accordance with the guidelines of the Institute for Laboratory Animal Research (Guide for the Care and Use of Laboratory Animals) and the US Public Health Service (Public Health Service Policy on Humane Care and Use of Laboratory Animals).

### Sample collection

Blood was collected for DNA extraction from a) privately owned crd3-affected and non-affected purebred GIT dogs; b) mixed breed dogs derived from GIT founders and maintained as a study colony as part of an NIH-sponsored project (EY006855) at the Retinal Disease Studies Facility (RDSF) in Kennett Square, PA; and c) from 80 privately owned pedigreed dogs from breeds not known to segregate crd3 (Table 1).

**Table 1:** Breeds tested for the presence of *ADAM9* crd3-mutation.

**Breed number**	**Breed**	**Number tested**
1	American Cocker Spaniel	5
2	American Eskimo	6
3	Australian Cattle Dog	5
4	Border Collie	5
5	Chesapeake Bay Retriever	5
6	Chinese Crested	5
7	Elkhound	3
8	English Springer Spaniel	5
9	Entlebucher Mountain Dog	5
10	Golden Retriever	5
11	Italian Greyhound	5
12	Labrador Retriever	5
13	Nova Scotia Duck Tolling	5
14	Siberian Husky	5
15	Tibetan Terrier	5
16	Portuguese Water Dog	6
**Total**		**80**

All dogs were homozygous normal.

### Phenotypic evaluation of study dogs

#### Clinical diagnosis

Diagnosis of *crd3* phenotype was based on ophthalmoscopic examination. In selected cases electroretinography was undertaken either to confirm the diagnosis, or to establish diagnosis before ophthalmoscopic evidence of disease, using methods described previously [10].

#### Morphologic evaluation

From selected colony dogs, eyes were enucleated post mortem and processed for morphologic evaluation using a triple-fixative protocol, essentially as described previously [11,12]. In brief, enucleated eyes were slit (5–10 mm) at the equator, and initially fixed whole by immersion in 3% glutaraldehyde-2% formaldehyde in 0.1 M Na cacodylate buffer (pH 7.2–7.4) at room temperature; after 5–10 min the anterior segment was removed by dissection with fine scissors, the vitreous was gently removed from the eyecup, and the eyecup was replaced into the same fixative, on ice. Eyecups remained in the first fixative at 4 °C for a minimum of 45 min and up to 24 h. Then the eyecup was transferred to the second fixative (freshly made 2% glutaraldehyde-1% osmium tetroxide, in 0.1 M Na cacodylate buffer, pH 7.2–7.4) on ice, for 45 min to 1 h. Next, the posterior segment was trimmed into four quadrants extending from the optic disc to the ora serrata, and the trimmed quadrants were separately placed into the third fixative (2% osmium tetroxide in 0.1 M Na cacodylate buffer) for 1 h at 4 °C, or on ice. The quadrants were then dehydrated in increasing concentrations of ethanol and embedded in an epoxy resin (PolyBed 812; Polyscience, Warrington, PA), sectioned at 1 µm (Supercut 2065 microtome; Leica, Deerfield, IL), stained with azure II (Sigma, Atlanta, GA) and methylene blue (Electron Microscopy Services, Hatfield, PA) and counterstained with paraphenylenediamine (Sigma). For each dog, 1 μm sections extending continuously from the optic disc to the ora serrata of superior, inferior, and temporal meridians were evaluated using a light microscope.

From selected epoxy embedded blocks, after an area of interest was first determined by light microscopy, a 1–1.5 mm square pyramid was trimmed; ultrathin sections (approximately 60 nm) were cut with a 3 mm diamond knife on a Reichert Ultracut 701701 ultra microtome (Reichert-LABTEC, München, Austria) and floated onto nickel thin bar grids. The grids were then stained for 20 min with aqueous 2% uranyl acetate, washed with degassed mQ water (Milli-Q-Synthesis A 10 System, Model=ZMQP6VFTl, Millipore Corporations, Burlington MA) and then stained with Sato modified lead stain for 7 min and washed as above. Grids were examined with an FEI Technai T20 Biotwin transmission electron microscope and images collected with a high-resolution thermoelectrically cooled Gatan Orius dual-scan CCD camera (Gatan, Inc.; Warrendale, PA).

### Whole-genome association study

#### Group design

Nineteen crd3-affected purebred GIT dogs were selected that were the least related to each other as possible, based on maximizing the number of grandparents contributing to the selected cases. Two GIT-derived, mixed-breed crd3-affected dogs were added to bring the total number of cases to 21. A total of 22 unaffected purebred GIT dogs were similarly selected as the least related to each other, but matched pairwise to the affected dogs (i.e., siblings or parents of affecteds).

#### Single nucleotide polymorphism genotyping

Samples were genotyped using Affymetrix Version 2 Canine single nucleotide polymorphism (SNP) chips and the standard Affymetrix GeneChip Mapping 250K Sty Assay protocol (Affymetrix Inc., Santa Clara, CA), using 250 ng genomic DNA. Genotypes were called using the MAGIC algorithm [13] on batched sets of the Affymetrix probe results files (CEL files).

#### Association analysis

Genotype calls were tested for association with disease phenotype using Fisher’s exact test to test for under/over-representation of the disease phenotype in one homozygote class versus the rest (i.e., versus a class pooling heterozygotes with homozygotes for the opposite allele). This test was done twice, pooling heterozygotes with either homozygote in turn. A Bonferroni correction for multiple tests was used to set the genome-wide significance threshold at -Log_10_(p)≥6.39 [0.05/(60,245*2)]. Genotype calls for chromosome 16 were assembled into haplotypes to identify the minimal linkage disequilibrium (LD) interval.

### Candidate genes evaluation

Candidate-gene screening was done by exon scanning (Table 2). *ADAM9* evaluation was done by amplification of overlapping fragments covering the complete coding region of the gene (Table 3), from normal and affected retinal cDNA (12 weeks and 13.4 weeks old, respectively) prepared with Reverse Trancriptase enzyme (ThermoScript, Invitrogen, Carlsbad, CA) and 2 μg retinal RNA following the manufacturer’s protocol. Twenty ng of DNA or cDNA was denatured at 95 °C for 2 min, and 35 cycles of 95 °C for 30 s, 58 °C for 30 s, and 72 °C for 1 min/1000 bp were performed in a thermal cycler (MJ Research, Watertown, MA). An additional final extension time of 5 min at 72 °C ensured full-length products. When necessary, PCRs were optimized by increasing the annealing temperature to up to 64 °C. The reactions were performed using GoTaq® Green Master Mix (Promega, Madison, WI) in a final volume of 25 μl. PCR products were run on 1.8% agarose and stained with ethidium bromide (2 μg/ml in a water bath). Single specific PCR products were extracted using the Qiagen PCR extraction kit (Qiagen) and eluted in 10 mM Tris–HCl (pH 7.5). Some products were extracted from the gel using a Qiagen gel extraction kit (Qiagen). A PCR product (200 ng/1000 bp) was mixed with 8 pmol of, either forward or reverse primer, and sequencing was performed using the Applied Biosystems Automated 3730 DNA analyzer (Applied Biosystems, Foster City, CA). Each PCR product was sequenced with the forward and reverse primer. Sequences were then analyzed and compared using Sequencher 4.2.2 software (Gene Codes Corp., Ann Arbor, MI).

**Table 2:** Primer pair sequences used to screen candidate genes within the minimal LD interval.

**Primer pair**	**Gene name**	**Forward primer name**	**Forward primer sequence**	**Reverse primer name**	**Reverse primer sequence**
1	*C8orf4*	C8orf4F1	AATCAACCCTGATAAGCCACAGAA	C8orf4R1	TAATGGGGTCCTTCAAATATCCAA
2	*C8orf4*	C8orf4F2	CAATCATCACAGACCAAGAGCAG	C8orf4R2	GAAGAAGAGGACGAAAGACAAGC
3	*C8orf4*	C8orf4F3	GTGTTGGGTCTCACAAACTCCTTC	C8orf4R3	ACATCCCTGCTCCATCCCTGA
4	*IDO1*	IDO1F1	AGTTTCTTTCCGACTTCCCCAAT	IDO1R1	ACTCTGCCTCTAATTTTGGCACTG
5	*IDO1*	IDO1F2	TGTGAAAAAGAGACAGGTGAGAGTG	IDO1R2	TAAAAATCAGGACGAGAGGATGGA
6	*IDO1*	IDO1F3	GGGTGATATGAACATTTTATGCCTCT	IDO1R3	AGGCTGTGTGCTTAGGTTGTAAGG
7	*IDO1*	IDO1F4	GATGGACGCACAAATAAATGACTG	IDO1R4	TACCCCTAAAGCAGAGAAGAAAGC
8	*IDO1*	IDO1F5	GCAGATAAACCACTGAAAAACTGAA	IDO1R5	TGATTGCTCTACTTATGCAAATGG
9	*IDO1*	IDO1F6	TCAGCCTAAAAATGAAGTGGAAATC	IDO1R6	CCAAGGACCCATCAGCAGTAAC
10	*IDO1*	IDO1F7	GGGGCTCTTGTTTCATTTTGTTTA	IDO1R7	CTACCCTTCCTGTCCATACTCCAG
11	*IDO1*	IDO1F8	CAGGATGGTGGTAAACTCATTTCC	IDO1R8	TACAAGGAAGGCACAGATATTGGA
12	*IDO1*	IDO1F9	TCCTTTCTTTTTCCCAAGTCATTTC	IDO1R9	TTGTCATCAGCAGCCAGTTGTT
13	*IDO1*	IDO1F10	TGATTTTCTTTTTCTCTTCCAACTGA	IDO1R10	CCAGCACTTTATCCTCTCACCTTT
14	*IDO1*	IDO1F11	GGAAGAAGAATAACGAAGCCGATT	IDO1R11	CAGATGAAATGCTGATGGGAAGTT
15	*TM2D2*	TM2D2F1	GGACCTGATTGGAGGAAGCAC	TM2D2R1	ATTGGTGAGAGCAGTGAGAAACCT
16	*TM2D2*	TM2D2F2	GACCAGAAACACTCCCAATGAAAG	TM2D2R2	GCTCAAAGAACAAAAGACAGGTTG
17	*TM2D2*	TM2D2F3	TCCACATTGACCATTCCCAAA	TM2D2R3	AAGGCTATTTTCACAGGATTTATGC
18	*TM2D2*	TM2D2F4	CACAGAGGAACTGCTACACCATCT	TM2D2R4	CACAAAAGGCACAAGGCTAAAAAT
19	*HTRA4*	HTRA4F1	AGAAAACTGTGGTGGAGGTGTGTT	HTRA4R1	GAGGAGGTAAAGGAGGCTGGATAG
20	*HTRA4*	HTRA4F2	CTCTACCCTTCCCACCCCTCCT	HTRA4R2	GCACGATGAAAACAGAAAGCAT
21	*HTRA4*	HTRA4F3	CGTGGTCTGTTGAGGTTTTTAGA	HTRA4R3	CGTGAAGGGGACAAGGACTATTTA
22	*HTRA4*	HTRA4F4	TGGCATAAATACACTGAAGGTGACA	HTRA4R4	GCAGACCCTAAAAAGACAGCAAGT
23	*HTRA4*	HTRA4_5F	TGTGTAGGCTCTGGAATAAATCTGA	HTRA4_5R	ACCTTACCTCTGCCCTTTCTCAT
24	*HTRA4*	HTRA4_6F	CACTTGGTGGTTGGTAAACATTGA	HTRA4_6R	CAGGCGACAGCATTGAGAAAA
25	*HTRA4*	HTRA4_7F	GGTTCAGTAGTTGAGCGATGGAAT	HTRA4_7R	AAGGTCAGCGTTAGGAAAACACAC
26	*HTRA4*	HTRA4_8F	TGAGCTGAGTGCATGGAAACTGT	HTRA4_8R	GTCATCCTGCGCCCTTTCTTC
27	*HTRA4*	HTRA4_9F	GTGGTGTGTGTGTGTATGGAGGTC	HTRA4_9R	AAGAAGGAGAGAAAACTGGGAACG
28	*HTRA4*	HTRA4_10F	GAGCGGAGTTGGGGAGAGACC	HTRA4_10R	GACCTTCAGTTATGTCGTGGGAGT
29	*HTRA4*	HTRA4_11F	GTGCTCTGGACCTCCCTGACTA	HTRA4_11R	TCCACATGCTTTAGATTCCCTGTT
30	*HTRA4*	HTRA4_12F	AACTGTGACCAGCTTGATGGAATC	HTRA4_12R	GCACATCTCAATCCACATATTTACCA
31	*HTRA4*	HTRA4_13F	ATGGTCTTTCTTTCCTCCCCTCTC	HTRA4_13R	CATCTTTGGCACATCTTCAATACAA
32	*LETM2*	LETF1	TTCTTCTTATTCCCCATCATTTGC	LETR1	CCTCTTCACAATCGCCCTAAAGTA
33	*LETM2*	LETF2	CAAGGTGACAGTTCTTTTCCTCTCA	LETR2	TGTCCAAGTCAGCCCAAGTCG
34	*LETM2*	LETF3	CCAGATGCCCCATGAAATACA	LETR3	TCCTTCCTAGTGGGTCCTAACTGA
35	*LETM2*	LETF4	ACTGGGACACACAACAAACGGTA	LETR4	GCAAAGTAGAAATCACAAAATGAGGTC
36	*LETM2*	LETF5	TTCTTTCCCCTTATCTGCTCTGTG	LETR5	ATCTGTGGAGACACCCCCGTTAT
37	*LETM2*	LETF6	AGGGATTTAGTGAGTGAAAAGCAA	LETR6	GTTTGGATGACAGGAAGAAGTGTG
38	*LETM2*	LETF7	CCAGAGCAGAAGGATGACACAAGT	LETR7	TTTACACAGTTTGGTGGGATGACA
39	*PPAPDC1B*	PPF1	GCCTTCCTCCGGTGCAGTTCC	PPR1	GTCTCTGGAGCCAAAATGGACATC
40	*PPAPDC1B*	PPF2	CTTTATTGCGGGGGTTGGTTTG	PPR2	CAGGAGGGGAGCGGAGGAAGG
41	*PPAPDC1B*	PPF3	GTCTAACCTTGCTCCTCTCGCTTG	PPR3	CAAACTCCTTCCTCTGTCCTTGAA
42	*PPAPDC1B*	PPF4	CGGATGAATGAGGGAGGTTCCTT	PPR4	TCAGGATAATGGAAAAATTGGGACA
43	*PPAPDC1B*	PPF5	TCCTACCAAGTATCAGAATGCCAGT	PPR5	CACCAGAGAAAGTAAACAGAGCCAAA
44	*DDHD2*	DDF1	GGGTGAGGGAGAGGGAGAAATAGA	DDR1	TCAAGAAAGAGAAGACCTGAGTTACAA
45	*DDHD2*	DDF2	GGGGAAGACGCAGGGATAACTT	FFR2	CGGAGCGGATGGAAACACAGTAA
46	*DDHD2*	DDF3	GAGGGCAGGAGCGTGTGAAAG	DDR3	ACAAACCCAGAATCCCAAGAAACA
47	*DDHD2*	DDF4	GTTGTGTGGCCCCCTTGAGATTAG	DDR4	AGGCTTTTCTGAGCTTCTGCTTGA
48	*DDHD2*	DDF5	TCCTCAAGCAGAAGCTCAGAAAAG	DDR5	TGAAAATACATGAAAAGGGATCAAGG
49	*DDHD2*	DDF6	AGCCTTTTATTTTTCTGGCTCTGA	DDR6	AAATGGTGGCAGTGGATACAA
50	*DDHD2*	DDF7	CACTGGTACGTTATGTGGGCATATT	DDR7	AATCCAAATCCATAGAAAAAGGTCA
51	*DDHD2*	DDF8	TGCAATCTTATTAACCTATTCATTGTGA	DDR8	TTCTACGGATTAAGGGCTTGTTCA
52	*DDHD2*	DDF9	TCATTGCTTCCCTGGTGATACATA	DDR9	TTCATCCATATCACTGATCAGAAAAC
53	*DDHD2*	DDF10	AAGCTTGAAGGCTAAGCTGAGTAA	DDR10	TGCTTCTATCAGCCAAGAATGACA
54	*DDHD2*	DDF11	AAAAATGTTGCTTCAACTAAAATTGC	DDR11	CTCTCCCACAAAGCAAAAATCACT
55	*DDHD2*	DDF12	GTAGTCTGGGAGGGAGAAGGAAGC	DDR12	TCACAAAGCAAAAGGATACAAGGA
56	*DDHD2*	DDF13	TTGCTCTTCATCATACTCTGCTATTG	DDR13	TGTTGCCCCATCACTTTCTGA
57	*DDHD2*	DDF14	GGATATTTTTATAACATAGCAGCTCCA	DDR14	AGGTGAGAACACAGGAGCTATCCA
58	*DDHD2*	DDF15	TTCTTGGCCTAATACACAGTTCCT	DDR15	AACAAAGTGAATGAGGTCCAGTCC
59	*DDHD2*	DDF16	CCCAGGACCCCAGGATTACAC	DDR16	TTCTGCTTCTCTTGACATCTTTCC
60	*DDHD2*	DDF17	AGAGGAGGAAAGTGCAGAAAGGTT	DDR17	ACCAGTGACCAAAGAACACCATTT
61	*DDHD2*	DDF18	CCTGTTCATTTGGTTTTTAGATTCC	DDR18	GTGGCTTTTCTGGGGGAGGAT
62	*DDHD2*	DDF19	TCCCCTTTTCAATCTGTGATAGGA		FOR SEQUENCING REACTION 48
63	*LSM1*	LSM1_F1	GAGAGTCGTGGGACGGAGGTC	LSM1_R1	GTCAACGCCGAACAGCCAGAA
64	*LSM1*	LSM1_F2	ATCCCTTGGTCCTTCCACTAAT	LSM1_R2	TCTATCGTTTGGCTCCCTCTACTG
65	*LSM1*	LSM1_F3	ATTCTTGTAACTGGTCCCCACCAT	LSM1_R3	GCTGGATGCAATGTGGAAAATATAC
66	*LSM1*	LSM1_F4	CGATCCCTACCACCACATTCATA	LSM1_R4	TCAGGATGTCACTTTCATTCAGTG
67	*LSM1*	LSM1_F5	AAACCCCGTCTGTCTCTCCAAC	LSM1_R5	GACCTCCGTCCCACGACTCTC
68	*STAR*	STAR1F	CTCTATCCTTGACCCCTTCCTCTG	STAR1R	GGTAGCCTCCGTGCCAATCTA
69	*STAR*	STARF2	GTCACTGCTGCCCTCCTCTCT	STARR2	CCCATCCCCTGTAGTCTGTGTATT
70	*STAR*	STARF3	CAAATACACAGACTACAGGGGATGG	STARR3	GACAGCAGAGGAACAGTGAGGAAC
71	*STAR*	STARF4	GTGGCAGGAAAGATTAGCAACTGT	STARR4	TGTTAGGGAAGAGAGGTTTTGAGG
72	*STAR*	STARF5	ATGAGGCAAGGCTGAGGTTTAG	STARR5	TTTTGAGGTGATGGAACAGTAGGC
73	*STAR*	STARF6	GCCTACTGTTCCATCACCTCAAA	STARR6	GCCCTCATTTTCTTGGTCCTAAA
74	*STAR*	STARF7	ATGGAAACAATGGGAGAGTGGAAC	STARR7	GCTGAAGGAAGAGACCAAGGAC
75	*EIF4BP1*	EIF4F1	GTTCTCACGGCAGGAACCGAAG	EIF4R1	GATGCGCCTTATTGCAGTCAC
76	*EIF4BP1*	EIF4F2	CCTAAGAGTGTATGAGGAAGAGGAAGC	EIF4R2	CACCAAAGGGGTCACAAAAGAC
77	*EIF4BP1*	EIF4F3	CACCACCAGCAAACACTGACA	EIF4R3	CAAAACAAAACAACCCTCCATTTC
78	*EIF4BP1*	EIF4F4	TAAAAGGACAGGCAGGGTGGCATA	EIF4R4	CAGGATAGGAAGATGAGTAACATTGC
79	*EIF4BP1*	EIF4F5	CTTTTGGGGTAAGGGGCAGAGT	EIF4R5	CATACAGGGACAGGAAATGGAAAC
80	*EIF4BP1*	EIF4F6	GCAGGAGGTTTGAGATGGCTTT	EIF4R6	GGGAAAATGCTTCAGGGACAAT
81	*EIF4BP1*	EIF4F7	ATGCCTCCTGTATTGGTCTGCTA	EIF4R7	ATCCATCCATCCTTTTCTGTGTGT
82	*EIF4BP1*	EIF4F8	CCTACTCTCCCTCCATTCTCTTTG	EIF4R8	GGTGGCTTTTATTTCCTCTCTTGG
83	*BRF2*	BRFF1	TAGACTGCAAAGAGGGGAAACAAC	BRFR1	AGGTGCTAGAAAAACAGACGAACG
84	*BRF2*	BRFF2	GACGGAAGGGTTATGGGTCAAT	BRFR2	GTGAAAACAGGAAAAGCAAGAGGA
85	*BRF2*	BRFF3	TCCAAAGAATGGTGGGTTGAAAT	BRFR3	GGGCACAAGAGAGGGAGACTACAT
86	*BRF2*	BRFF4	TGTACTCCTTTTCCTTGCTTTGGA	BRFR4	GTGTGAGTGCTCCCCTGGATG
87	*BRF2*	BRFF5	GCTGCTACCTTCCTGGCTTGG	BRFR5	GATAGGGGGTGGGGGACAGAT
88	*GPR124*	GPR124F1	CTCTTGACCCCACCTGTCTGAA	GPR124R1	GGAACTCACCTCCCATCTCTGG
89	*GPR124*	GPR124F2	CCTTTACCGAGAAGAAACCTCCAG	GPR124R2	CTGACCCTGTCCTGCTTTGTG
90	*GPR124*	GPR124F3	CACTCTCCCCACCACTCTCCT	GPR124R3	ATCCACTTTCCCCACCCTCAG
91	*GPR124*	GPR124F4	AGGGCTGAGGAGAATCCAGTTC	GPR124R4	GAGAGTGGGAAGGCAATGGTG
92	*GPR124*	GPR124F5	GTATCTCTTCCCACCCAAGGAC	GPR124R5	TGCCCACTACTCAATAACACAAGG
93	*GPR124*	GPR124F6	AAACCCACCTGTTCCTCTCTTGT	GPR124R6	CGAGCCAGACCCTTTATGACTTAG
94	*GPR124*	GPR124F7	GAATGTACTTGTGTCCTTCGCTCA	GPR124R7	CTTTCTCCATCTCCTGCTTTCTCC
95	*GPR124*	GPR124F8	CACGAAGTTAGAAAGAAGGTGGAG	GPR124R8	CAGAACAGAGGGGCAGCAGAG
96	*GPR124*	GPR124F9	GCGTTAGAAGTGGTGTTAGAAGTGG	GPR124R9	GCTGCCCAAAGACAGGAGTGT
97	*GPR124*	GPR124F10	AGGAGTGTGTAGGGGGACAAATCT	GPR124R10	CGGAGTGTGTCTGTAACCTTTTTG
98	*GPR124*	GPR124F11	ACCACTCATTTGGCATTTGGAAT	GPR124R11	GCCGCCTTTCTGTAGTTCTCG
99	*GPR124*	GPR124F12	CCAGATGGACTAGGGGCTAAAGTT	GPR124R12	CAGTGGAGATGGGGCCTTTTA
100	*GPR124*	GPR124F13	GTGGGAGTAGGGGTGGTAAGAAGT	GPR124R13	CAGGGCAGGCTCAGTAGGTTC
101	*GPR124*	GPR124F14	TAAATGGGAAAGAGGATGGGACAG	GPR124R14	AGTAGCCAGAAGGGAACCTGAGTC
102	*GPR124*	GPR124F15	CACCCTTCATCCACTGCCTGT	GPR124R15	CCCCTCGCACACCTGACTCTG
103	*GPR124*	GPR124F16	CCCTGCAAGCTCACCAACCTG	GPR124R16	GTCGTCGTACTTGGCTCCCTTG
104	*GPR124*	GPR124F17	CTCAACGCAGCCAGTCTGAAC	GPR124R17	GAGAGGTGGGGAGTACCTATGGAG
105	*ERLIN2*	ERLF1	GCATAAAGGGCAATCCCAAATAG	ERLR1	GATGGCTTTCTTGGTCCTGAG
106	*ERLIN2*	ERLF2	GTTGTTGGCGTAGCCTTGTGTT	ERLR2	GTGGGTACAAACTAGCGGAGCAG
107	*ERLIN2*	ERLF3	GAAACTTCTCATCTGTTAAGGATTGC	ERLR3	TGTTGGTAAACACCCCAAACC
108	*ERLIN2*	ERLF4	CTGTGCTTGATGGTTTTCAGAGTG	ERLR4	CAGAAGGCAGGGAACTTGGTG
109	*ERLIN2*	ERLF5	ATGAACTCTGCTGCTCCTTTGCT	ERLR5	CTGCCTTCCAGTCCTCTGATTTG
110	*ERLIN2*	ERLF6	CTAGAAGTGGGACAGGGACCATAA	ERLR6	AAAAGTGATTCTGACGATTTCTCAA
111	*ERLIN2*	ERLF7	GGTGCCTGCCTTCTCTTTAGC	ERLR7	CAACACACTCGCTCCATCTGAC
112	*ERLIN2*	ERLF8	TTTATGGCTCATGCCAAAGAAGAT	ERLR8	AATGGGGAGACTCAAAACTCACTG
113	*ERLIN2*	ERLF9	TGTGACTTAGGAGGGAGGTTAGGG	ERLR9	ATCTGCTGGGCGAGTGAATGT
114	*ERLIN2*	ERLF10	TACTCACTACGGGGACTCTCAAGG	ERLR10	CTACAACTGAATGCCACCAACAGA
115	*ERLIN2*	ERLF11	GGAGGGGAAGAGGAGTAAGCAT	ERLR11	CTAAGGGAAGGGGCAATACCTGT
116	*ERLIN2*	ERLF12	GCAAACCCATTAGTACCCTGTCAC	ERLR12	TGCTTACCTGAATAAGACCCCAAA
117	*ERLIN2*	ERLF13	CTGTTGCCCTTCTCTGTTCAAATA	ERLR13	GGAAACTGCTGACTTCCATGATTT

**Table 3:** Canine *ADAM9* primer sequences, locations, and product sizes observed by retinal RT–PCR from crd3-affected and normal dogs.

**Primer pair**	**Forward primer name**	**Forward primer sequence**	**Forward primer location**	**Reverse primer name**	**Reverse primer sequence**	**Reverse primer location**	**Observed size in normal (bp)**	**Observed size in affected (bp)**
1	ADAM9_cDNA_F3	GTTGAGTGGAACCTGCGGAATCT	5′ UTR	ADAM9_cDNA_R3	GGCTCTTCTTCTTCATAGTTTGTGG	Exon 6	610	610
2	ADAM9_cDNA_F2	ATTCATCCATTGCTCTCAGCGACT	Exon 5	ADAM9_cDNA_R2	TACAAAGTTCCCCAACACATCACC	Exon 9	482	482
3	ADAM9_cDNA_F4	TGTCCTGCCACAGACCCGATA	Exon 7	ADAM9_cDNA_R4	TGGAACATCACATTCGTTGGTTTT	Exon 14	832	832
4	ADAM9_cDNA_F1	GAGTGTGAATCGGACCCTTGTTGT	Exon 13	ADAM9_cDNA_R1	CATTTGGTGCCTTTACTGGGAGTC	Exon 16	497	**NO**
5	ADAM9_cDNA_F6	CTGTATTTGGAATCGTGCCTGCTA	Exon 16	ADAM9_cDNA_R6	TACTTTCGGTTGTGGTGGAGGTG	Exon 22	647	**NO**
6	ADAM9_cDNA_F5	TCAGATGGCAAAAATCAAGCAAAA	Exon 20	ADAM9_cDNA_R5	ATCCATGTTCGGTGCATTAACTTC	3′ UTR	541	541
7	ADAM9_cDNA_F7	TGTCAGCCAGATGTTTTTATTCAGA	Exon 14	ADAM9_cDNA_R7	GTCCACACTTCCTCCGTATCCTTT	Exon 18	573	**283**
8	ADAM9_cDNA_F8	TTGTGGTTTCTCTGGCAATGA	Exon 15	ADAM9_cDNA_R8	CACTTCCTCCGTATCCTTTAGTCTCA	Exon 18	389	**NO**
9				ADAM9_cDNA_exon1R	GATATAAGACACCTGTTCTGAATAGG	Exon 1		

NO=not observed.

### *ADAM9* mutation identification

To further characterize canine *ADAM9*, 33 primer pairs were designed to amplify and sequence the genomic interval between exon 14 and exon 17 from one normal and two affected dogs (Table 4).

**Table 4:** Primer information and product sizes observed in crd3-affected and normal dogs, used to identify the canine crd3 mutation.

**Primer pair**	**Forward primer name**	**Forward primer sequence**	**Forward primer location**	**Reverse Primer name**	**Reverse primer sequence**	**Reverse primer location**	**Observed size in normal (bp)**	**Observed size in affected (bp)**
**A. Initial sequencing of the region.**								
1	deletion_F1	CGAGGAAAAACCAACGAATGTGAT	Exon 14	deletion_R1	gagcaagcaatgaaaatagcagacc	Intron 14	521	521
2	deletion_F2	cctacctaataaccccatcccttgg	Intron 14	deletion_R2	tcagaaccctatccttttggcttg	Intron 14	627	627
3	deletion_F3	ctctgtgagtgaggtggacttagc	Intron 14	deletion_R3	ccaaggaatgaaacataaaatggaa	Intron 14	691	NO
4	deletion_F4	gagccagtagggagaggaacatca	Intron 14	deletion_R4	aaagacgcagaggaccacacaac	Intron 14	691	NO
5	deletion_F5	caagaatagtttgctaccttgtcagc	Intron 14	deletion_R5	ataccctggatcaactgccacatt	Intron 14	612	NO
6	deletion_F6	tgggtgttatgttcttcttgctca	Intron 15	deletion_R6	gaatgtgtagaggggcagagg	Intron 15	610	NO
7	deletion_F7	tcttagcagagccagagagccttc	Intron 15	deletion_R7	cagtttatccttccttcaacattcacg	Intron 15	622	NO
8	deletion_F8	atttggtggtcaacattcattggt	Intron 15	deletion_R8	cattcaaaacaactggaagcaggt	Intron 15	611	NO
9	deletion_F9	acgctgttgtgcagatcgtactgt	Intron 15	deletion_R9	aaccgtgagatgaaacatttgtgg	Intron 15	999	NO
10	deletion_F10	tctaaaatggaggaagtgtgaactaca	Intron 15	deletion_R10	ttcttggcttgggctaactct	Intron 15	602	NO
11	deletion_F11	tgtctgtccacgctctctgctatc	Intron 16	deletion_R11	tctctctacctctccctctgtttcca	Intron 16	688	NO
12	deletion_F12	ttgttacccgccataccccttgt	Intron 16	deletion_R12	ggagcaaagatgaaaaatacaggaa	Intron 16	759	NO
13	deletion_F13	ggaacaaacgcctctctcagtctt	Intron 16	deletion_R13	accagtagtccaaagggtccaggt	Intron 16	795	NO
14	deletion_F14	gaaatggtggggtggtagacaaga	Intron 16	deletion_R14	tactggggaaagatgagggttttt	Intron 16	774	NO
15	deletion_F15	tgactgtgaggggaagtgaagagtt	Intron 16	deletion_R15	cgttgaatgatggtatttggagatga	Intron 16	805	NO
16	deletion_F16	tgtcttctgttttggttgccagtg	Intron 16	deletion_R16	ccccgaactcatcccttactttct	Intron 16	835	NO
17	deletion_F17	acacatccccattccaactttcag	Intron 16	deletion_R17	accatcaactctcctggctctcag	Intron 16	796	796
18	deletion_F18	agcaccctcacaaacattcaga	Intron 16	deletion_R18	tcctctcaggcttttaccattatctt	Intron 16	798	798
19	deletion_F19	gaaaggaagtgtttgctgtagggaaa	Intron 16	deletion_R19	tctggatgaggtgagagtgaatgg	Intron 16	729	729
20	deletion_F20	aagattgaccgcttttcaccta	Intron 16	deletion_R20	ctacagatggctttgggcagtatg	Intron 16	718	718
21	deletion_F21	atccaggggaaatgaaaacaggag	Intron 16	deletion_R21	ggctgagagagcagaccagattgt	Intron 16	787	787
22	deletion_F22	tgagaagacaaatgaggggcactt	Intron 16	deletion_R22	tcaaaccaggcaatcaaacacctt	Intron 16	822	822
23	deletion_F23	cagaaggtgtttgattgcctggtt	Intron 16	deletion_R23	ttttgtttcccacagcatttttga	Intron 16	769	769
24	deletion_F24	tgctgatttctcccattattacca	Intron 16	deletion_R24	cacagttcctacaccaccaccaac	Intron 16	739	739
25	deletion_F25	aacttcatctaccctccttcacttg	Intron 16	deletion_R25	agtctcacctacctcactgggaat	Intron 17	752	752
**B. Refined sequencing of the region.**								
26	deletion_F26	caagccaaaaggatagggttctga	Intron 14	deletion_R26	tcactccacaggtaaaaagccaaga	Intron 14	459	459
27	deletion_F27	tgactgaacccaggaagagagttg	Intron 14	deletion_R27	tgaatgaacaggcgaaaaagagag	Intron 14	462	462
28	deletion_F28	acctggattgggtttctttagg	Intron 14	deletion_R28	gcccgtggagtgggacataacta	Intron 14 and Intron 16	487	487
29	deletion_F29	ctggagcaatggggctggata	Intron 14	deletion_R29	aaaccaaaagcaataaataccacaa	Intron 14 and Intron 16	649	649
30	deletion_F30	atcagtcgttgagggtgacattga	Intron 14 and Intron 16	deletion_R30	ccgtggaaaagaaaaatcagacct	Intron 14 and Intron 16	421	421
31	deletion_F31	gggaaggatgggagaatgagagta	Intron 14 and Intron 16	deletion_R31	tcaaaggagcaatcggaaaagtct	Intron 16	476	476
32	deletion_F32	aaagggaaagggagggacagact	Intron 16	deletion_R32	tgtgagataaaggaaaataaagttgga	Intron 16	658	658
33	deletion_F33	cacaggctaacttttgctccatgt	Intron 16	deletion_R33	tgagtcttccttgccagtagaagc	Intron 16	479	479
34	deletion_F29	ctggagcaatggggctggata	Intron 14	deletion_R31	tcaaaggagcaatcggaaaagtct	Intron 16	NO	1,515

A. Initial analysis of the region. B. Refined analysis of the region. NO=not observed: PCR failed to amplify a fragment.

### Mutation screening

To identify crd3-affected, -carrier, and -normal dogs, a multiplex PCR was designed to amplify the normal and mutated alleles in one PCR reaction. One primer pair, specific to the normal allele, was located within the deleted sequence, and one primer pair specific to the mutant allele flanked the deletion.

### RNA expression

RNA was extracted from a 13.4-weeks-old crd3-affected retina and from 8.6-, 10.4-, and 12-weeks-old normal retinas, as well as 7.7- and 15.7-weeks-old normal brains and a 22.1-weeks-old normal spleen. For Northern analysis, 10 μg of total RNA was mixed with 10 μg/ml ethidium bromide and 3X gel loading buffer (Ambion, Austin, TX) in a final volume of 10 μl, heated at 65 °C for 10 min, chilled on ice for 2–3 min, and loaded on a 1% agarose-formaldehyde denaturing gel; 3 μg of 0.24–9.5 kb RNA ladder was used as a size marker (Invitrogen). The gel ran with continuously circulating 1X Mops running buffer (Ambion) for 16 h at 21 V. After three 5-min washes in DEPC-treated water, 20 min in 0.05 N NaOH, and a 15-min soak in 10X SSC, transfer to a nylon-based membrane (GeneScreen Plus; NEN Life Science, Boston, MA) was done with 10X SSC buffer using a standard protocol. Full transfer was confirmed by exposing the gel to UV light. The membrane was washed in 2X SSC for 2 min, and RNA was cross-linked to the membrane (exposure was 0.12 J/cm2; Stratalinker UV Crosslinker; Stratagene, La Jolla, CA). An ADAM9 probe was produced by amplification of normal retinal cDNA using primers located on the 5′ UTR and exon 6, to produce a probe 610 bp long. The product was then cloned (TOPO TA cloning kit, Invitrogen), and the probe was labeled with [α-^32^P]dCTP using a RadPrime DNA Labeling System (Invitrogen) according to the manufacturer's protocol, then used for blot hybridization. Hybridization was performed using Ultrahyb solution (Ambion, Austin, TX) following the manufacturer’s protocol, and the blot was exposed to X-ray film at −70 °C for 4 day with two intensifying screens. Loading control was achieved by hybridizing a canine-specific beta-actin probe to the membranes under the same conditions and exposing it to X-ray film for 2 h.

## Results

### Phenotypic evaluation

Clinically, GIT dogs affected by crd3 were diagnosable by ophthalmoscopic examination as young as 3 years of age, although some affected dogs did not show lesions until much older. With one exception, none of the dogs diagnosed as clinically non-affected when examined at ages over 8 years have since proven to be homozygous for the *ADAM9* mutation. The exceptional case was a 10 years-old GIT that presented with an unusual geographic pattern of pigmentary disturbance in the fundus of both eyes, but with no overt evidence of the retinal thinning or vascular attenuation expected in a crd3-affected dog of this age. Other than this dog, disease was recognized ophthalmoscopically in all crd3-affected dogs by 5 years of age. Two distinctly different clinical phenotypes, however, were observed. In the majority of cases, disease first became apparent as a subtle but generalized alteration in the fundus, as is typically seen in many other forms of late-onset hereditary retinal degeneration in dogs. In these cases, subtle but generalized hyperreflectivity of the tapetal fundus, and retinal vascular attenuation, were the predominant signs of early disease, and these changes worsened appreciably within 12–24 months of initial observation. In a minority of cases, the initial ophthalmoscopic change detected was confined to the area centralis (mid-temporal tapetal fundus), as a discrete, distinctly hyperreflective lesion, with no accompanying ophthalmoscopic evidence of generalized retinal disease. In these dogs, the central lesion remained unchanged for at least 12 months after initial detection, but over a longer period (2–4 years), it did eventually progress to generalized retinal involvement.

**Figure 1:**
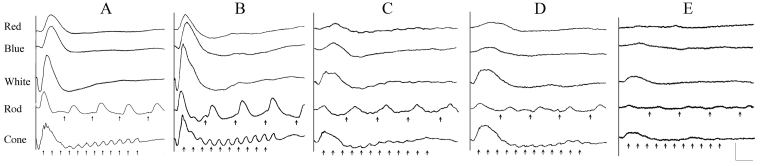
Electroretinograms of normal and affected dogs. Electroretinograms (ERGs) from a 7 weeks old normal dog (**A**), a 12 weeks old dog affected with canine cone-rod dystrophy 3 (crd3; **B**), a crd3-affected dog aged 1.2 years (**C**), a 2 years old crd3-affected dog (**D**), and a 4.9 years old crd3-affected dog (**E**). Each vertical panel presents electroretinogram (ERG) responses to a red flash, a blue flash, a white flash, 5 Hz low-intensity white flashes (Rod), and 30 Hz high-intensity white light flicker (Cone). Short vertical arrows under the Rod and Cone flicker responses indicate the onset of the flickering light stimuli. Red and White traces represent mixed rod-cone responses, Blue and Rod traces are rod-specific, and Cone traces are cone-specific. Responses of the 12-weeks-old crd3-affected dog appear normal (**B**), but by 15 months of age, cone dysfunction is detected as reduced 30 Hz flicker responses (**C**), and is followed at later ages by continued deterioration of both cone and rod responses (**D**, **E**). At all ages, the loss of cone function is more prominent than that of rods. Vertical calibration bar=100 µV; horizontal=200 ms for rod flicker; and other responses are 100 ms.

The electroretinogram (ERG) of a 12-weeks-old, crd3-affected dog (Figure 1B) was indistinguishable from normal (Figure 1A), showing normal rod- and cone-mediated responses to light stimuli. At about 15 months of age, ERG dysfunction was detected as reduced 30 Hz cone flicker responses (Figure 1C), and at later ages by continued deterioration of both cone and rod responses (Figure 1D,E). At all ages, the loss of cone function was more marked than that of rods, hence the designation of the disorder as a cone-rod dystrophy.

### Morphology

**Figure 2:**
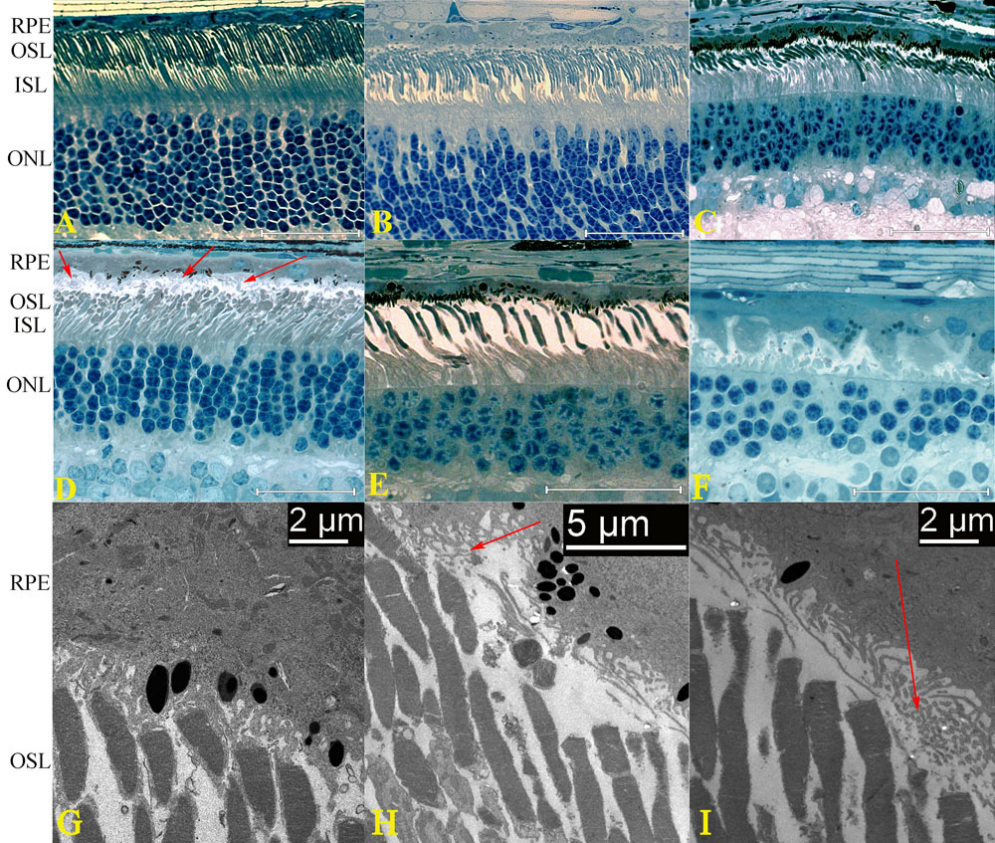
Light- and electron-microscopic retinal morphology in normal and canine cone-rod dystrophy 3 (crd3) affected dogs. In the retina of a 27-weeks-old non-affected dog (**A**), the outer nuclear layer (ONL) comprises approximately 10 rows of rod nuclei and a single distal row of cone nuclei. The inner and outer segments of the photoreceptors (IS, OS) are of consistent proportions, tightly aligned, and parallel, and the distal OS tips are in close proximity to the apical membrane of the retinal pigment epithelium (RPE). In retinas of 4.7- and 13.4-weeks-old crd3-affected dogs (**B**, **C**), rod and cone IS and OS lack the tightly packed highly parallel organization of a normal photoreceptor layer, and the distal OS tips appear to be more distant from the RPE apical membrane than in normal dogs. In the retina of an 18-weeks-old crd3-affected dog (**D**), IS and OS are disarrayed and disorganized, and a distinct gap is observed between the RPE and the OS (arrows). The retinas of 26-weeks- and 5 years-old crd3-affected dogs (**E**, **F**) exhibit continued photoreceptor degeneration as evidenced by loss of cone and rod IS, OS, and nuclei. Electron micrograph of the retina of a 27-weeks-old nonaffected dog (**G**) shows that the microvilli from the RPE apical membrane extend to invest the photoreceptor OS. Electron micrographs of the retina of a 13.4-weeks-old crd3-affected dog (**H**, **I**) show that the RPE apical microvilli form a tangled flattened mat that does not extend to invest the photoreceptor OS (arrows).

By light microscopy, at 4.7 weeks of age, the rod and cone inner (IS) and outer segments (OS) of the crd3-affected retina lacked the tightly packed, highly parallel organization of a normal photoreceptor layer (Figure 2). At 4.7, 13.4, and 18 weeks of age (Figure 2B,C,D), an apparent gap was consistently observed between the distal ends of the photoreceptor OS and the retinal pigment epithelium (RPE; Figure 2D, arrows); IS and OS appeared disarrayed and disorganized; and structural abnormalities of cone IS and OS were at least as or more severe than those of rods. At later ages, distinct thinning and reduction of cell numbers in the ONL was observed (Figure 2 E,F). At 5 years of age (Figure 2F), cones and rods were severely reduced in number, with only a few nuclei remaining in the ONL, most of which appeared rod-like.

By electron microscopy, in the crd3-affected retina, the apical microvilli of the RPE showed very little of the normal investiture of rod and cone outer segments (Figure 2 H,I). Mostly these microvilli formed a flattened, entangled mat between the RPE cell bodies and the distal OS tips of the photoreceptors. This mat appeared to correspond to the gap seen between the RPE and the OSL by light microscopy.

### Whole-genome association analysis

Of the approximately 127,000 loci represented on the Affymetrix vs2 SNP chip, 60,245 passed the quality control incorporated into the MAGIC algorithm. One affected sample was contaminated and was excluded from the analysis. Comparison of two duplicated samples, to assess the consistency of the MAGIC program, found 98.4% of the calls to be identical in the first duplicate, and 93.3% in the second duplicate, for an average of 95.8%, and most mismatched calls were between an unclustered call and a defined call.

**Figure 3:**
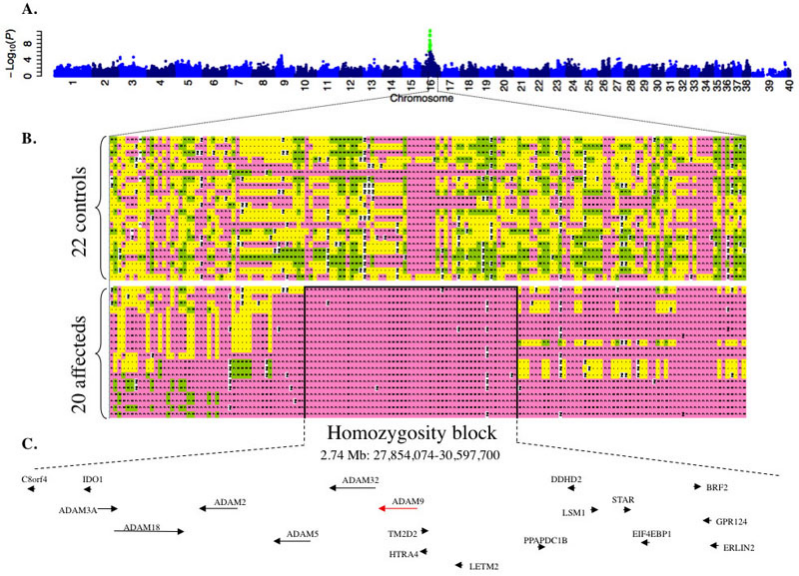
Results of genome-wide association study in canine cone-rod dystrophy 3. The statistical signal (y-axis, negative Log10 [Fisher exact test 2-tailed probability]) for association between canine single-nucleotide polymorphism (SNP) genotype and canine cone-rod dystrophy 3 (crd3) phenotype, plotted against SNP chromosomal location (**A**), demonstrates a distinct peak on canine chromosome 16 (CFA16). Green dots are SNPs for which the association signal exceeded the Bonferroni threshold for genome-wide significance. Chromosome X is represented by the numbers 39 and 40. Homozygosity analysis of SNP genotypes (**B**), in the region of CFA16 yielding the peak association signal, reveals heterozygosity throughout the interval in 21 nonaffected control dogs, and demonstrates a 2.74 Mb homozygosity block in 20 crd3-affected dogs. Genotypes are color coded as follows: pink and green represent the major and minor genotypes observed in affecteds, respectively; yellow is heterozygous; and white is missing data. Black lines border the 2.74 Mb homozygosity block. Refseq genes screened as potential positional candidates for crd3 in the present study (arrowheads), and ADAM family genes identified within the crd3 minimal linkage disequilibrium interval (arrows) are indicated with annotation and order consistent with the CanFam2 canine genome assembly (**C**, not drawn to scale).

Fisher Exact analysis, comparing genotypes of cases to controls, identified an association signal on CFA16 (Figure 3) extending over 6 Mb and including 28 SNPs that exceeded the Bonferroni corrected significance threshold (-Log_10_(p) range=6.39–10.09, Table 5). The peak p-value (-Log_10_(p)=10.09) was shared by six SNPs comprising an interval of approximately 4.4 Mb (CFA16: 22,690,750–27,122,415). All affected dogs were homozygous at these SNP loci, suggesting a recessive mode of inheritance. With that in mind, all genotype calls on CFA16 were aligned to identify a homozygosity block, that is, where all genotype calls for all affected dogs were homozygous, between positions 27,854,074 and 30,597,700–a 2.74 Mb interval (Figure 3; Appendix 1). The block of homozygosity was not observed in controls except for one dog (dog number 17 in Appendix 1). This dog, when first examined at 10 years of age, had retinal lesions that were regarded as incompatible with a diagnosis of crd3, but in retrospect were likely to represent an unusually delayed and mild form of crd3 disease. Thus, this homozygosity block represents the region of absolute linkage disequilibrium (LD) of crd3 in the GIT population examined

**Table 5:** Genome wide association study results

	**A. Sort by -Log10(p)**		**B. Sort by location**	
Number	Location	-Log_10_(p)	Location	-Log_10_(p)
1	**28600467**	**10.09**	25601422	6.39
2	**28926606**	**10.09**	27844483	7.76
3	**29299541**	**10.09**	27845657	8.88
4	**30342464**	**10.09**	27854074	7.76
5	**30480010**	**10.09**	28286469	9.34
6	29748980	10.09	28301327	9.03
7	28286469	9.34	28592790	9.03
8	30509498	9.34	**28600467**	**10.09**
9	28301327	9.03	**28926606**	**10.09**
10	28592790	9.03	**29299541**	**10.09**
11	27845657	8.88	29495970	8.16
12	29949196	8.46	29543182	8.16
13	29967119	8.46	**29748980**	**10.09**
14	30022978	8.46	29949196	8.46
15	30031243	8.46	29967119	8.46
16	30348569	8.19	30022978	8.46
17	29495970	8.16	30031243	8.46
18	29543182	8.16	30039251	7.67
19	31638702	8.12	**30342464**	**10.09**
20	30473690	7.93	30348569	8.19
21	27844483	7.76	30473690	7.93
22	27854074	7.76	**30480010**	**10.09**
23	30039251	7.67	30509498	9.34
24	31810810	7.28	31638702	8.12
25	31664333	6.97	31664333	6.97
26	31793958	6.97	31793958	6.97
27	31823035	6.61	31810810	7.28
28	25601422	6.39	31823035	6.61

Single Nucleotide Polymorphisms that passed Bonferroni correction, location on chromosome 16, and -Log_10_(p) values sort by -Log_10_(p) values (**A**) and by location (**B**). Bolded are the single nucleotide polymorphisms (SNPs) with the highest -Log_10_(p) values.

### Homozygosity analysis

To evaluate the power of association mapping by homozygosity, we analyzed genotype calls from just the 20 affected dogs (59,408 SNP calls from autosomal chromosomes) to identify homozygosity blocks greater than 1.0 Mb. Five such blocks were identified where all 20 affected dogs were homozygous for the same allele, and 10 blocks where 19 of the 20 were homozygous for the same allele (Table 6).

**Table 6:** Homozygosity evaluation.

**Chromosome**	**Size of block >1.0 Mb**	
	19/20 dogs homozygous to same allele	20/20 dogs homozygous to same allele
1	1.02	-
12	1.25	-
12	3.57	-
12	1.19	-
14	1.4	1.4
**16**	**3.14**	**2.74**
20	1.26	1.26
21	1.94	1.94
26	1.33	1.33
38	1.2	-
Total	10	5

Homozygosity analysis was done on genotype calls for crd3- affected dogs (n=20). Blocks of homozygosity for the same allele were measured, and those greater than 1.0 Mb were recorded. In bold is the homozygosity block that bears the mutation for crd3.

### Candidate gene screening

The crd3 minimal LD interval (CFA16: 27,854,074 - 30,597,700) was identified as homologous to part of human chromosome 8p11, although the gene order is rearranged between human and dog (data not shown). Although CORD9, a human autosomal recessive cone-rod dystrophy, had previously been mapped to this human interval [14], the CORD9 gene had not yet been identified at the time of the current study. Comparison of genes common to the CORD9 mapped interval and the crd3 LD interval identified 31 potential candidate genes. Ten of these were completely screened (chromosome 8 open reading frame 4 [*C8orf4*]*,* indoleamine 2,3-dioxygenase 1 [*IDO1*]*,* TM2 domain containing 2 [*TM2D2*]*,* leucine zipper-EF-hand containing transmembrane protein 2 [*LETM2*]*,* phosphatidic acid phosphatase type 2 domain containing 1B [*PPAPDC1B*]*,* DDHD domain containing 2 [*DDHD2*]*, LSM1* homolog, U6 small nuclear RNA associated (*S. cerevisiae*)**[*LSM1*]*,* steroidogenic acute regulatory protein [*STAR*]*,* eukaryotic translation initiation factor 4E binding protein 1 [*EIF4EBP1*]*,* and ER lipid raft associated 2 [*ERLIN2*]) and three (HtrA serine peptidase 4 [*HTRA4*]*,* BRF2, subunit of RNA polymerase III transcription initiation factor, BRF1-like [*BRF2*], and G protein-coupled receptor 124 [*GPR124*]) were partially screened, by exon scanning (Figure 2C; Table 2). All 10 were excluded from association with crd3.

### *ADAM9* evaluation

When a mutation in *ADAM9* was established as associated with CORD9 [9], we evaluated its role in crd3-affected dogs. The human *ADAM9* gene (NM_003816.2) has 22 exons and encodes an 819-amino acid protein. Blasting this sequence against CanFam2 identified 21 of the predicted canine exons but failed to identify the complete sequence of exon 4. This sequence was identified completely in the CanFam1 assembly. Six primer pairs were designed from CanFam2 to amplify overlapping retinal cDNA fragments of the complete canine *ADAM9* coding sequence (Table 3, primer pairs 1 to 6). From normal retinas, all six primer-pairs each amplified a single product and alignment of the overlapping sequences established a 2,781 bp canine *ADAM9* cDNA sequence (accession number HM590630) comprising 29 bp of 5′ UTR, 2,460 bp of coding region, and 292 bp of 3′ UTR.

From affected retinas, primer pairs 4 (exon 13 to 16) and 5 (exon 16 to 22) both failed to amplify a product. Subsequently, amplification with primer pair 7 (exon 14 to 18) gave 573 bp and 283 bp product from normal and affected dogs, respectively. Primer pair 8 (exon 15 to 18) failed to amplify cDNA from the affected dog, but yielded a 389 bp product from normal dogs. Sequence analysis of the 283 bp crd3-affected amplicon from primer pair 7 showed that exons 15 and 16 are absent from affected cDNA.

**Figure 4:**
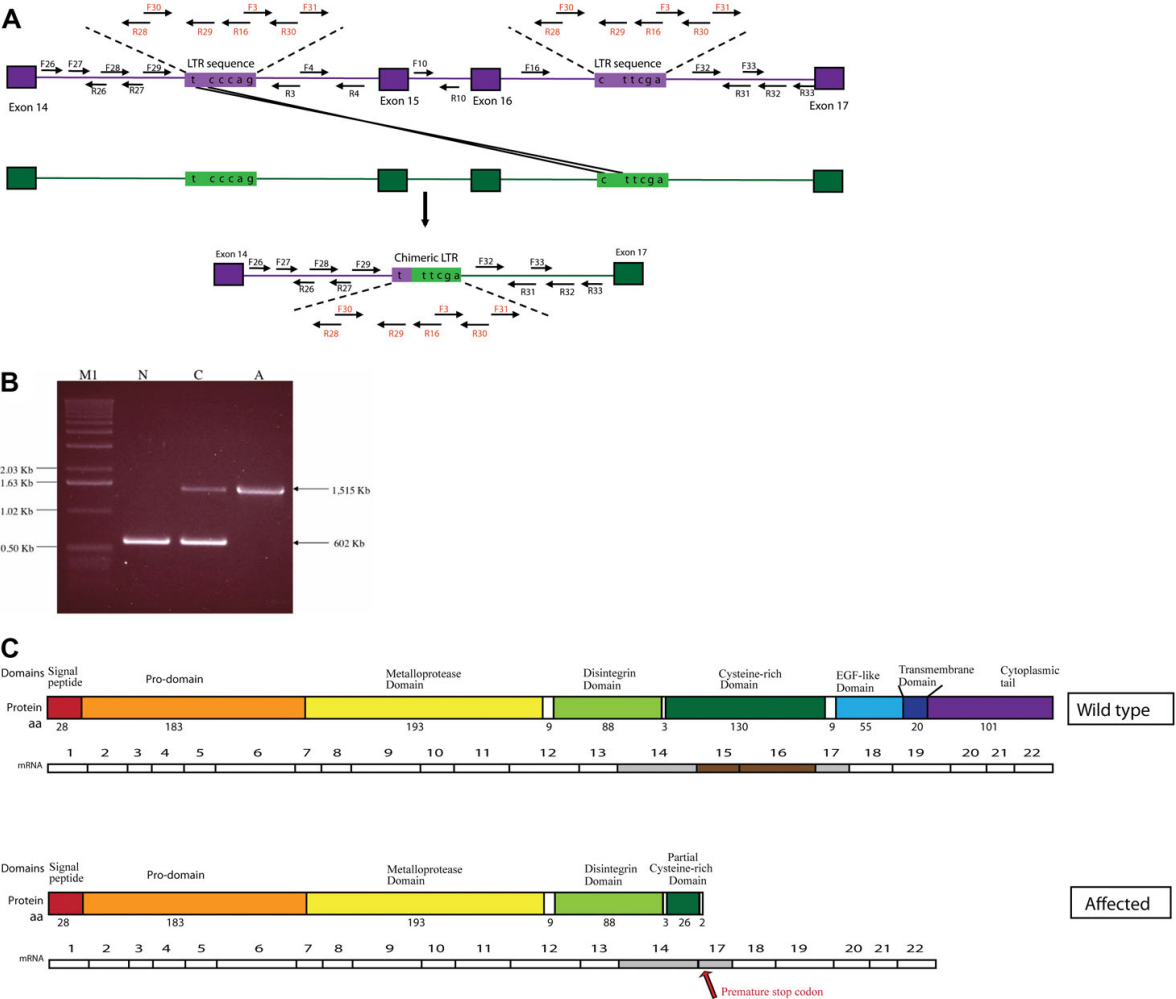
*ADAM9* mutation in canine cone-rod dystrophy 3 (crd3) affected dogs. A schematic drawing, of part of the canine *ADAM9* genomic sequence, aligning the normal and crd3 mutant alleles suggests a possible mutation mechanism. Bordered square boxes represent exons 14–17, lines between are introns; unbordered rectangular boxes represent long-terminal repeat sequences (LTR). Within the LTR are the nucleotides identified as single nucleotide polymorphisms (SNPs). A suggested mechanism of unbalanced recombination is illustrated, resulting in the deletion of part of intron 14, all of exon 15, intron 15, exon 16, and part of intron 16, as well as the formation of a single chimeric LTR. Arrows represent the location of primers used to identify the mutation. Primer pair F29/R31 amplifies the mutant allele and results in a 1,515 bp PCR product from genomic DNA. Primer pair F10/R10 amplifies the normal allele and results in a 602 bp PCR product from genomic DNA. **B**: Gel electrophoresis of multiplex PCR reaction identifies ADAM9 alleles from normal (N), crd3-carrier (C), and crd3-affected (A) dogs. The lower, 602 bp band is the normal allele, amplified by primer pair F10/R10 located within intron 15, which is deleted in the affected allele. The upper 1,515 bp band is the mutant allele, amplified by primer pair F29/R31, which flanks the >23 kb sequence deleted in the affecteds. Both bands are present in the heterozygous carrier dog. The normal and crd3 mutant canine *ADAM9* transcripts, and their corresponding predicted translation products, are aligned (**C**) to illustrate their differences schematically. The protein domains represented are those predicted by Swiss-Prot for the human ADAM9 protein. Exons 15 and 16 are missing from the mutant transcript, and a premature stop codon is introduced (arrow). The mutant protein translated from this transcript is predicted to be truncated, lacking the last 287 amino acids of the C-terminus, part of the cysteine-rich domain, the complete epidermal growth factor (EGF)-like domain, the transmembrane domain, and the cytoplasmic tail.

The genomic interval between exon 14 and exon 16 was partially amplified and sequenced from one normal and two affected dogs (Table 4). This interval spans over 40 Kb in CanFam2 (CFA16: 29,384,764–29,425,539). Initial screening used 25 primer pairs, evenly distributed (Table 4). Primer pairs 3 to 16 failed to amplify DNA in the two affected dogs. This suggested a deletion of at least 21 Kb, between intron 14 and intron 16. To refine the deletion points, PCR was done using an additional eight primer pairs, amplifying overlapping fragments (Table 4, F26/R26–F33/R33). Surprisingly, all eight primer pairs amplified fragments from the affected dog that were identical in size to those from the normal dog. Critical examination of the CanFam2 sequence revealed an LTR sequence in intron 14 (29,422,135–29,423,412) and another in intron 16 (29,398,914–29,400,191), with 99.5% identity (6 SNPs in 1,278 bp). That suggested that unbalanced recombination between the two LTRs might have led to a deletion of more than 23 Kb, resulting in a single fused copy of the LTR and deletion of exon 15 and 16 from the genome (Figure 4A). To test this hypothesis, PCR was undertaken with primers F29 (on intron 14, upstream from the first LTR) and R31 (on intron 16, downstream from the second LTR). This distance is >24 Kb in the normal genomic DNA, and in the proposed scenario should be only 1,515 bp in the affected dogs. The PCR failed to amplify DNA in the normal dog, and amplified the predicted size product from affected ones. The sequence of this product revealed a deletion of 23,221 bp between the LTR in intron 14 and the LTR in intron 16. This deletion included part of intron 14, all of exon 15, intron 15 and exon 16, and part of intron 16. The SNPs in the LTRs suggest that the unbalanced recombination took place between the first and the second of these SNPs, and resulted in a chimeric, single LTR (Figure 4A).

### Population mutation screening

Multiplex PCR was designed to amplify the normal and mutated alleles of canine ADAM9 (Table 7, Figure 4A,B). Primer pair 10F/10R, located within intron 15, will only amplify a 602 bp-long fragment from a normal chromosome. The affected allele is detected using primers F29/R31, which flank the deletion. All dogs included in the GWAS were genotyped. All 20 affected dogs were homozygous for the mutation; five control dogs were carriers (dogs 7, 9, 10, 12, and 13 in Appendix 1, of which 10 and 13 were obligate heterozygotes because they produced affected dogs); and 16 controls were homozygous normal. Dog number 17 in the control group, whose genotype in the minimal LD region was identical to that of affected dogs, was homozygous for the mutation, confirming this dog’s affected status.

**Table 7:** Genomic PCR primers. Primer pairs, sequences, location and observed product sizes in normal, *crd3*-carrier, and *crd3*-affected DNA, used in multiplex analysis. NO=not observed.

**Primer pair**	**Forward primer name**	**Forward primer sequence**	**Forward primer location**	**Reverse primer name**	**Reverse primer sequence**	**Reverse primer location**	**Observed size from normal dog (bp)**	**Observed size from crd3-carrier dog (bp)**	**Observed size from crd3-affected dog (bp)**
1	F10	tctaaaatggaggaagtgtgaactaca	Intron 15	R10	ttcttggcttgggctaactct	Intron 15	602	602	NO
2	F29	ctggagcaatggggctggata	Intron 14	R31	tcaaaggagcaatcggaaaagtct	Intron 16	NO (>24 Kb)	1515	1515

Thirty-six further purebred GIT dogs, not part of the GWAS, were screened for the mutation. Ten of these that had been diagnosed as affected were homozygous for the mutation; 24 non-affected dogs were either homozygous normal, or heterozygous; and two dogs that were clinically non-affected when examined at young ages (1.2 and 2 years old) were homozygous for the mutation, and presumably will become affected at a later age. A subset of crd3-colony dogs was genotyped as well, and showed segregation of the *ADAM9* mutation with the disease (data not shown). The *ADAM9* mutant allele was not found in 80 dogs from 16 other different breeds (Table 1).

The association between the six most significant SNPs identified from the GWAS was further analyzed by comparing the genotype for each SNP with that at the *ADAM9* mutant locus, and calculating the correlation coefficients. Two SNPs (rs22468640 and rs22463503, located 798 Kb and 472 Kb upstream from the mutation, respectively) were in complete linkage disequilibrium with the mutation, with r=1, while the other four had correlation coefficients between 0.929 and 0.976 (data not shown).

### *ADAM9* mutation: in silico protein analysis

The 2,781 bp canine *ADAM9* mRNA (accession number HM590630, Appendix 2) is predicted to encode an 819-amino acid protein, and comparison to the human ADAM9 protein database (Swiss-Prot) suggests that this protein includes a signal peptide (amino acids 1–28, translated from exon 1); a pro-domain (amino acids 29–211, exon 2–exon 7); a metalloprotease domain (amino acids 212–404, exon 7-exon 12); a disintegrin domain (amino acids 414–501, exon 12-exon 14); a cysteine-rich domain (amino acids 505–634, exon 14-exon 17); an EGF-like domain (amino acids 644–698, exon 17-exon 19); and a transmembrane domain (amino acids 698–718, exon 19). Amino acids 29–697 are extracellular, 698–718 are transmembrane, and 719–819 represent the cytoplasmic tail (Figure 4C; Appendix 2).

Amplification of the *ADAM9* coding region from crd3-affected retina identified a 2,491 bp-long, transcript that included all exons except 15 and 16, which are deleted from genomic DNA (Appendix 2). The mutation causes a frame shift, and results in a premature stop codon at base number 6 of exon 17. The mutant protein translated from this transcript is predicted to be truncated and to lack the last 287 amino acids of the C-terminus. That would remove part of the cysteine-rich domain, the complete EGF-like domain, the transmembrane domain, and the cytoplasmic tail (Figure 4C; Appendix 2).

### RNA expression profile

**Figure 5:**
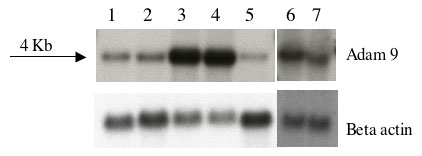
*ADAM9* RNA expression profile in normal and crd3-affected retinas. Northern blot, RNA expression of *ADAM9* in frontal lobe, brain of 15.7 weeks-old normal dog (Lane 1); brain of 7.7 weeks-old normal dog (Lane 2); retina of 10.4-weeks old normal dog (Lane 3); retina of 8.6-weeks old normal dog (Lane 4); spleen of 22.1-weeks-old normal dog (Lane 5); retina of 12-weeks old normal dog (Lane 6); and retina of 13.4-weeks-old crd3-affected dog (Lane 7). A single band is observed at approximately 4.0 Kb. *ADAM9* is highly expressed in retinas not affected with crd3 (lanes 3, 4, and 6), and at lower levels in brain and spleen (lanes 1, 2, and 5). In crd3-affected retina (lane 7), the mutant allele is observed as a slightly smaller band, and its level of expression appears slightly reduced compared to the normal.

Full-length RNA expression was evaluated by northern blot assay to determine the *ADAM9* mRNA transcript size and absolute level of expression in crd3-affected retinas, compared to normal ones. Northern blot analysis showed one transcript of about 4.0 Kb, highly expressed in non-affected retinas, and in lower levels in the brain and spleen (Figure 5). In the affected retinas, a smaller-size band was observed, which corresponded to the mutated allele, and the level of expression seemed slightly reduced, compared to normal.

## Discussion

Recent advances in canine genomics have increasingly highlighted and exploited the wealth of hereditary traits that the domesticated dog provides. Such traits range from morphology and behavior, through a panoply of genetic diseases and disease susceptibilities, and extend to broader aspects of population and genomic evolution [15–19]. One specific and highly productive focus within this broader field has been into the numerous hereditary retinal diseases that segregate in specific canine populations. Numerous genes causally associated with these retinal diseases have been identified [20,21] providing novel insights into the structural and functional mechanisms involved in normal and diseased retinas [22], and a suite of canine models for preclinical evaluation of potential therapies for comparable human diseases [23–26].

Canine population structure comprises multiple discrete, essentially closed populations; the specific breeds of dog lend themselves to genetic studies in much the same way as do the isolated human populations that geneticists have appreciated for many years [27–30]. Genome-wide association studies (GWAS) of Mendelian and complex traits in isolated human populations typically use platforms yielding genotypes for more than 500,000 informative SNPs [31–33]. GWAS efficiency is facilitated by the large LD blocks in these human isolate populations. In particular, young isolates with relatively few founders show particularly extensive LD with few gaps [31].

The division of the canine population structure into multiply isolated subsets, each of fairly recent origin and characterized by significant inbreeding and a restricted number of founders, permits a GWAS to be undertaken with a relatively lower number of cases and controls, and a much less dense set of SNP loci [6,7,32–35] than has typically been needed in human populations. In the present study, a GWAS identified the locus responsible for cone-rod dystrophy in the Glen of Imaal Terriers (crd3), using a carefully selected set of 20 affected and 22 non-affected dogs. Evaluation of a homozygosity approach suggested that just genotyping a large group of affected dogs could be adopted in autosomal recessive diseases, where the tradeoff for using fewer samples comes in the form of a few false positive regions of homozygosity. Nonetheless, this could be useful where the availability of cases and controls is severely restricted.

Positional candidate gene analysis identified a 23,221 bp deletion within the canine *ADAM9* gene, leading to a loss of exons 15 and 16, a premature stop codon, and a predicted protein truncated at its c-terminus. This establishes crd3 as a true ortholog of human CORD9, in which four distinct *ADAM9* mutations have been found [9]. The latter mutations are either nonsense mutations or frame-shift mutations leading to nonsense change, all within the pro-domain or the metalloprotease domain. The canine mutation resembles the human in that it causes a frame-shift and a nonsense change, which would lead to a truncated protein.

Initially, the *Adam9^−/−^* mouse was reported as having no evident major abnormalities during development or adult life [36], but subsequent reevaluation identified electroretinographic abnormalities, suggesting a progressive degeneration affecting both rods and cones [9]. Histologically, the retinas of these mice showed an abnormal gap between the photoreceptor outer segment distal tips and the RPE apical membrane, which electron microscopy revealed to be a failure of the RPE apical processes to invest the OS; instead, they formed a flattened distorted mat between the OS and the RPE [9].

The light microscopic and ultrastructural morphological abnormalities of crd3-affected canine retinas reported herein are clearly consistent with those observed by Parry et al. in the *Adam9^−/−^* mouse [9]. As in the mouse, the canine *ADAM9* mutant retina exhibits disarray, disorganization, and progressive degeneration of the photoreceptors. Unique to these two orthologous diseases, and presumptively therefore involved in human CORD9, is the failure of RPE apical microvilli to invest the photoreceptors. This strongly suggests that this structural abnormality reflects the absence of a critical function served by *ADAM9*.

ADAM proteases form a still-growing family of transmembrane proteins. The human genome contains 25 *ADAM* genes, of which four appear to be pseudo-genes (*ADAM1*, *ADAM3*, *ADAM5*, and *ADAM6*) and nine are expressed in the retina (*ADAM9*, *ADAM10*, *ADAM11*, *ADAM12*, *ADAM15*, *ADAM17*, *ADAM19*, *ADAM23*, and *ADAM33*; UniGene). In contrast, the mouse and rat have 37 and 34 *ADAM* genes, respectively, many of which are specifically expressed in testis. In the canine genome, 30 *ADAM* genes are predicted (Genome), with the five additional genes, compared to human, apparently corresponding to mouse genes specifically expressed in testis. Most of the genes in this family lack a functional protease domain, and appear to code primarily for adhesion proteins. ADAM9 is one of a few that have both adhesion and proteolysis functions [37–39]. Most ADAMs feature a significant overlap of substrate specificities, which may explain why inactivation of individual ADAMs only rarely causes major phenotypes. With that said, careful evaluation is needed, as the phenotype alteration is sometimes overlooked [36].

Comparison of the predicted canine, human, and mouse ADAM9 proteins shows that the human and canine sequences are more similar to each other than are human and mouse sequences (92.6% and 78.4%, respectively). The differences reside predominantly in the cytoplasmic tail, where only 14.7% of the mouse amino acids are identical to the human, compared to a 93.1% identity for dog amino acids. Furthermore, the mouse cytoplasmic tail has 26 additional residues that are absent from both the human and canine proteins.

Identification of this *ADAM9* mutation in crd3 provides a potentially important canine model for CORD9. The opportunity to undertake synergistic studies among orthologous murine and canine models and human patients has been very useful both for advancing knowledge of the disease mechanism in numerous disorders and for developing potential therapies. CORD9-affected human patients suffer childhood-onset visual acuity impairment, progressing over decades to major loss of central and then peripheral visual function [14]. Thus, disease severity clearly establishes a basis for considering genetic therapy. In the canine model, the retina is not fully developed until 8 weeks of age, the cone and rods are largely intact as late as 12 weeks postnatally, and the ERG shows normal responses up to one year of age. Thus, there is not only a prima facie case for therapeutic intervention based on disease severity, but also a significantly long-term window of opportunity.

## Appendix 1. Genotype calls from 20 crd3-affected cases and 22 control dogs, in a 6.1 Mb interval on canine chromosome 16.

The interval includes all SNPs yielding an association signal exceeding the Bonferroni corrected threshold for genome wide significance. Columns=dogs, rows=SNP loci. For each locus, homozygosity for the most frequent allele observed in cases is color-coded in pink for all dogs (i.e., cases and controls); green indicates homozygosity for the minor allele observed in cases; yellow indicates heterozygosity; and white (–)=no call. The crd3-absolute LD interval is boxed. Control dog 17 proved to be affected with an atypical form of crd3 (see text). To access the data, click or select the words “Appendix 1.” This will initiate the download of a compressed (pdf) archive that contains the file.

## Appendix 2. Canine *ADAM9* mRNA and predicted protein.

A. Canine *ADAM9*, normal allele. A1. *ADAM9* mRNA sequence from normal dogs. Upper case indicates coding sequence, boxed are the first methionine and the stop codon. In bold are exons 15 and 16 that are missing in the affected dog. A2. Canine ADAM9, predicted protein from a normal dog. The protein is 819 amino- acids long. Features in the protein are as follow: Red sequence: signal peptide- 1–28 aa (translated from exon 1); Grey sequence: 29–211 aa (translated from exon 2- exon 7); Orange sequence: Metalloprotease domain: 212–404 aa (translated from exon 7-exon 12); Blue sequence: Disintegrin domain: 414–501 aa (translated from exon 12-exon 14); Pink sequence: Cystein rich domain: 505–634 aa (translated from exon 14-exon 17); Brown sequence: EGF-like domain: 644–698 aa (translated from exon 17-exon 19); Green sequence: Transmembrane domain: 698–718 aa (translated from exon 19). Amino acids 29–697: extracellular, amino acids 698–718: transmembrane; amino acids 719–819: cytoplasmic. B. Canine *ADAM9*, crd3 mutant allele. B1. Sequence of crd3 mutant canine *ADAM9* mRNA. Boxed are the first methionine and the premature stop codon. ◊ indicates the location of exons 15 and 16 missing in the affecteds. Underlined is the stop codon in a normal dog. B2. Canine ADAM9 predicted mutant protein product. Colored are the domains. The last two amino acids are not present in the normal protein. The predicted protein is 532 amino- acid long. To access the data, click or select the words “Appendix 2.” This will initiate the download of a compressed (pdf) archive that contains the file.
